# Diversity Patterns of Insect Assemblages in *Tilia cordata* Stands in Lithuanian Protected Areas: A Two-Year Study Indicating Modest Support for Pollinator Guilds

**DOI:** 10.3390/insects17040360

**Published:** 2026-03-25

**Authors:** Jūratė Lynikienė, Artūras Gedminas, Rita Verbylaitė, Virgilijus Baliuckas, Valeriia Mishcherikova, Vytautas Suchockas

**Affiliations:** Lithuanian Research Centre for Agriculture and Forestry, Institute of Forestry, Kėdainiai Distr., LT-58344 Akademija, Lithuania; arturas.gedminas@lammc.lt (A.G.); rita.verbylaite@lammc.lt (R.V.); virgilijus.baliuckas@lammc.lt (V.B.); valeriia.mishcherikova@lammc.lt (V.M.); vytautas.suchockas@lammc.lt (V.S.)

**Keywords:** *Tilia cordata*, small-leaved lime, genetic conservation units (GCUs), insect diversity, non-bee pollinators, monitoring

## Abstract

Insects underpin key ecosystem services. Yet tree-associated insect communities remain comparatively poorly documented, particularly in temperate forests. This study aimed to characterize the diversity and abundance of insect assemblages associated with the predominantly insect-pollinated forest tree *Tilia cordata* Mill. in protected areas in Lithuania, and to assess the occurrence of known and putative pollinator groups within these assemblages. We quantified insect assemblages associated with *Tilia cordata* using two sampling methods but did not directly measure pollination effectiveness (e.g., pollen loads, visitation rates to flowers, or fruit/seed set). Consequently, our inferences refer to the presence and composition of potential pollinators rather than demonstrated pollination function or realized pollination services. Fieldwork was conducted over two years in six protected *T. cordata* sites in Lithuania using two complementary sampling methods: net sampling and sticky traps. Sampling was structured into three observation periods corresponding to *T. cordata* phenology: pre-flowering (I), flowering (II) and post-flowering (III). In total, 207 insect taxa from 15 orders were recorded by net sampling and 86 taxa from 11 orders by sticky traps. Net sampling showed significantly higher diversity (Shannon *H* = 3.81) than sticky traps (*H* = 2.10). Hemiptera, Coleoptera and Diptera were the most common groups, and most taxa occurred at low to moderate abundances, with only a few species showing local dominance in specific periods or sites. Taxa documented in the literature as significant pollinators were consistently present but at low relative abundances (about 5–10% in total). Insect assemblage composition and species proportions varied among phenological periods and between years, with no clear, consistent peak in overall insect abundance or diversity associated specifically with the *T. cordata* flowering phase. These findings indicate that *T. cordata* stands in protected areas harbor diverse insect assemblages typical of temperate deciduous and mixed forest habitats and include a broad spectrum of non-bees and other potential pollinators. Therefore, we did not detect a distinct peak in insect abundance or species richness during the *T. cordata* flowering period, indicating that flowering did not coincide with a pronounced maximum in pollinator-related insect activity. However, the quantitative patterns observed suggest that, in this context, *T. cordata* provides only modest support for pollinator guilds, and its role is better interpreted as one component of wider forest insect diversity rather than as a primary driver of pollination services.

## Simple Summary

Many insects around the world are declining because of habitat loss, intensive farming, pesticides, diseases, parasites, and climate change. In European forests and semi-natural woodlands, the small-leaved lime tree (*Tilia cordata*) can provide abundant nectar and pollen during the summer and may therefore support a variety of flower-visiting insects. In this study, we examined the insect diversity that visits *T. cordata* and how they might contribute to pollination. Over two years, we sampled insects using two methods: hand netting and yellow sticky traps. Hand netting captured more species and showed higher insect diversity, while sticky traps caught larger numbers of individual insects. Our results showed that *T. cordata* stands host diverse insect assemblages dominated by groups such as true bugs, beetles, and flies. Known or likely pollinators were consistently present, but usually at low relative abundances, and many common taxa were not clearly linked to flower visitation. Overall, *T. cordata* stands in protected forests support structurally diverse insect communities that include, but are not dominated by, pollinator guilds.

## 1. Introduction

Tree canopy-dwelling insect assemblages are highly diverse and encompass multiple trophic and functional guilds, most notably herbivorous species, predators, parasites and pollinators. Yet insect abundance and diversity are declining due to habitat fragmentation and land-use change, intensive agriculture, pesticides and climate change [1]. Current species extinction rates are estimated to be 100–1000 times higher than background levels due to anthropogenic impacts, with insects among the most affected groups. Approximately 40% of invertebrate pollinator species, particularly bees and butterflies, are facing extinction [2]. Plant–insect interactions are therefore of critical importance: without pollinators, many plants cannot reproduce, and without floral resources, numerous animal populations would decline, with cascading effects across ecosystems [3]. Human health, agriculture and natural resources are influenced by insect ecology and diversity [4].

Over recent decades, substantial declines have been documented in managed and wild pollinators, with the strongest evidence from Europe and North America, where habitat loss, landscape simplification, and agricultural intensification are key drivers [5,6]. Wild pollinators are essential for plant reproductive success and ecosystem functioning. Cross-pollination enhances genetic diversity, seed production, and offspring vigor [7,8]. Pollen limitation is widespread and is expected to intensify as pollinator diversity declines [7,9]. In some regions, long-term changes in pollinator communities have already shifted plant communities toward more self- or wind-pollinated species, illustrating the ecological consequences of reduced pollination services [5].

Some types of forest may be very important for pollinators in various landscape contexts, particularly if they provide essential resources for forest-specific taxa [10].

Within temperate Europe, lime trees (*Tilia* spp.) are important nectar producers, offering floral resources during the first half of summer. *Tilia cordata* Mill., the small-leaved lime, is a predominantly insect-pollinated forest tree [11]. It occurs mainly in mixed stands with other broadleaved species and spruce, rarely forming pure stands [12].

Its prolonged flowering, rich nectar secretion (approximately 2–3 mg per flower), and broad distribution across natural and semi-natural forests suggest that *T. cordata* can provide seasonally important floral resources for a range of arthropod assemblages, including wild pollinators and other flower-visiting insects whose roles are not yet fully characterized [13,14,15,16]. The chemistry of *T. cordata* nectar and its potential effects on pollinator physiology further underscore the complexity of these interactions [17].

Although bees are widely regarded as the principal pollinators in temperate systems, many non-bee insects also contribute substantially. Diverse taxa within Diptera, Coleoptera, Lepidoptera, and non-bee Hymenoptera transport pollen while foraging for nectar and/or pollen, providing complementary pollination services [18]. Dipterans, in particular, influence floral ecology, reproductive outcomes, and network stability [19]. Across temperate forests, beetles, flies, moths, butterflies, and some wasps can act as flower visitors and, in certain systems, as effective pollinators [18,20,21,22,23,24,25]

Functional diversity among these groups differing in phenology, foraging behavior, and environmental sensitivity can enhance ecological resilience and stabilize pollination networks [18,25]. The abundant, accessible flowers of *T. cordata* are likely to attract a broad spectrum of insect visitors, some of which may act as pollinators alongside bees.

Despite the ecological and socio-economic importance of *Tilia cordata* as a nectar and honeydew providing tree, comprehensive assessments of its flower-associated insect visitor communities in forest interiors remain scarce. More broadly, synthesis work on pollinators and pollination highlights that most empirical research has focused on crops and herbaceous vegetation, whereas tree-focused studies of insect–flower interactions in forest ecosystems are comparatively underrepresented [26]. Available evidence on tree–insect associations often derive from urban contexts [27], whereas limes can be important floral resources shaping insect assemblages in various environments [28,29].

In natural temperate forests, *T. cordata* typically occurs as scattered individuals or irregular tree groups within mixed stands rather than forming pure stands, which complicates targeted ecological investigation and may lead to underrepresentation of tree-associated flower visitors in entomological research [30].

This study addresses these gaps by characterizing the diversity and abundance of canopy-dwelling insect assemblages associated with *T. cordata* in protected forest stands in Lithuania and by examining the occurrence of known and putative pollinator groups within these assemblages. By comparing sites, sampling periods linked to *T. cordata* phenology, and using two sampling methods (net sampling and sticky traps), we assess how different approaches capture flower-visiting and other canopy-associated insects. Rather than quantifying pollination effectiveness, our aim is to provide baseline information on insect assemblage structure in *T. cordata* stands and to explore the extent to which these assemblages include taxa with potential relevance for pollination within temperate forest landscapes.

## 2. Materials and Methods

### 2.1. Study Sites

**Figure 1:**
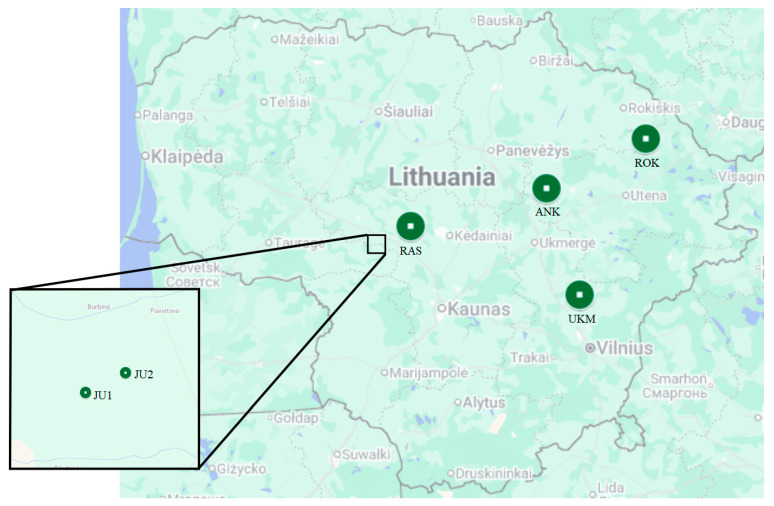
Map of Lithuania showing the location of study sites at each *Tilia cordata* Genetic Conservation Unit (GCU).

Permanent study sites were established within all six *T. cordata* Genetic Conservation Units (GCUs) at six different locations in Lithuania, with one study site per GCU (Figure 1).

Two GCUs are forest seed stands (Raseiniai regional branch of State Forest Enterprise (r. b. of SFE), code 17LSM003 (RAS) and Anykščiai r. b. of SFE, code 46LSM002 (ANK)), one genetic reserve (Rokiškis r. b. of SFE, code 55LGD003 (ROK)), and three State genetic reserves (two in Jurbarkas r. b. of SFE, codes 23LGD001 (JU1) and 23LGD002 (JU2), and one in Ukmergė r. b. of SFE, code 58LGD004 (UKM)). Information on the GCUs and main stand characteristics is in Table 1. The study stands were relatively similar in structure and site conditions. At all study sites *T. cordata* trees prevailed and composed 40–70% of all tree species; trees were mature (age ranged from 79 to 138 years); mean height ranged from 25.6 to 29.3 m; mean diameter ranged from 28.8 to 41.7 cm; *T. cordata* trees grow mainly in temporarily waterlogged mineral soils of moderate or high fertility, predominantly with an *aegopodiosa* vegetation type (Table 1). All study sites were located within forest interiors and were surrounded by at least 1–2 km of mixed forest, with the exception of the RAS site, where agricultural fields approximately 1 km in width extended from north to south along the forest strip. Adjacent to the ANK site, there was a clear-cut area with abundant *Tilia* and *Populus* regeneration. To assess the surrounding areas of the GCU sites, we used forest cadastre data from the Lithuanian geoportal site (https://www.geoportal.lt/geoportal/, accessed on 1 March 2026).

At each study site, two insect sampling methods were applied to compare insect assemblages associated with (i) the lower canopy of *T. cordata* trees at the forest edge (net sampling) and (ii) the middle canopy of *T. cordata* trees growing in the forest interior (yellow sticky traps). Given this, any differences observed between these methods may be attributable not solely to the sampling techniques themselves but also to vertical stratification of insect assemblages and contrasting habitat conditions (edge versus interior). Insect monitoring with both methods was conducted in 2023 and 2024. For each year, sampling was stratified into three observation periods corresponding to *T. cordata* phenology: pre-flowering (I), flowering (II) and post-flowering (III).

### 2.2. Net Sampling

Net sampling was used to survey insect assemblages associated with the lower canopy (1–2 m above ground) of *T. cordata* at forest edges. At each study site, 50 sweeps were made with an entomological net around approximately 20 *T. cordata* branches, covering a crown area of about 30 m^2^. Sampling was conducted on dry days. Collected insects were transferred into glass containers with cotton wool soaked in 99.2% chloroform for 15 min., then sieved to remove plant material and placed into plastic boxes. For each site and observation period (pre-flowering, flowering, post-flowering), 6 samples were collected over 2 monthly sampling dates in May–June (pre-flowering), June–July (flowering) and July–August (post-flowering) (6 *T. cordata* GCUs × 1 study site per GCU × 6 times × 2 years). Samples were transported to the laboratory and dried at room temperature for 20 days.

### 2.3. Sticky Traps

Canopy-level insect assemblages in the forest interior were sampled using yellow sticky traps. Traps consisted of 20 × 20 cm yellow plastic sheets covered on both sides with a transparent polyethylene sheet (20 × 40 cm) coated with non-drying glue (Pestifix, “Flora”, Talinn, Estonia). At each study site, four *T. cordata* trees within a radius of ca. 10 m from one another were selected, and one trap was installed in the canopy tree at a height of 10–15 m above the ground, resulting in 24 traps per monitoring year (6 study sites × 4 sticky traps). A Big Shot^®^ slingshot (Notch Equipment, Greensboro, NC, USA) with a weighted throwline was used to rig ropes in the canopy to raise the traps. Traps were placed as high as possible and within approximately 3 m of the sun-exposed crown edge (south-facing and/or upper crown), in order to maximize and standardize canopy catches while limiting long-distance visibility. The exact position of traps in the crown was further constrained by the availability of suitable branches for rope rigging.

Traps were lowered down once per month, and sticky polyethylene sheets with captured insects were replaced, providing three sampling time points that corresponded to the pre-flowering (May–June), flowering (June–July), and post-flowering (July–August) periods of *T. cordata*. Sticky sheets with captured insects were transported to the laboratory on the same day and stored at 5 °C until taxonomic identification.

### 2.4. Insect Identification and Functional Classification

Many specimens were identified to the species level, whereas others were identified to the genus, family or order only. Identification was performed under a Zeiss Stemi 2000-C microscope (Oberkochen, Germany) based on morphological characters and using standard identification keys [33,34,35,36], supplemented by online databases [37,38,39] and consultation with a taxonomic specialist (see Acknowledgments). In the sticky trap samples, some insect individuals could not be reliably identified because of missing body parts or extensive coverage by glue [40]. These specimens were retained in the dataset at the coarsest taxonomic level to which they could be assigned (typically order or family), and their abundances were included in community-level analyses (e.g., diversity indices and multivariate ordinations). However, they were not used in any interpretations that required species-level resolution. Insects identified only to the order level were classified as having an unknown role in pollination. Based on published information on feeding habits and flower-visiting behavior, each taxon with at least family-level identification was assigned to one of four broad functional categories with respect to pollination: (i) significant pollinators, (ii) potential pollinators (including flower visitors, pollinivores, nectarivores and honeydew feeders), (iii) insects with unknown role in pollination, and (iv) non-pollinators: taxa not typically associated with flowers. Functional categories were assigned at the species level whenever species-level information on feeding and flower-visiting behavior was available; otherwise, assignment followed genus- or family-level evidence, applying the most conservative category when taxa encompassed mixed or uncertain ecological roles. For taxa whose documented ecology included both flower-visiting and non-flower-related habits, we adopted a conservative approach and classified them either as potential pollinators or with unknown or negligible pollination effectiveness or as non-pollinators, unless clear evidence of effective pollination was available. Classification was primarily based on the CABI Compendium [41] and additional sources retrieved via Google Scholar. The functional assignment of each identified taxon is listed in Supplementary Tables S1 and S2.

### 2.5. Statistical Analysis

The Shannon diversity index [42] was used to characterize the diversity of insect assemblages associated with *T. cordata* at each site, for each method and year. Differences in Shannon diversity between sites, sampling methods, and years were tested using the nonparametric Mann–Whitney–Wilcoxon test in Minitab v.19.2 (Minitab^®^ Inc., Pennsylvania State University, State College, PA, USA). The number of unique and shared insect taxa between sampling methods (net sampling vs. sticky traps), study years and phenological periods of *T. cordata* (pre-flowering, flowering and post-flowering) were illustrated using Venn diagrams produced with an online tool [43] (accessed on 20 April 2024). Differences in the relative abundance and species richness of insect taxa between study sites and methods were analyzed using the Kruskal–Wallis H test in XLSTAT (Addinsoft, New York, NY, USA).

The composition of insect assemblages was examined using nonmetric multidimensional scaling (NMDS) based on the Bray–Curtis dissimilarities performed in R version 4.0.5 (R Core team, Vienna, Austria) (accessed on 10 June 2024). The NMDS ordinations were visualized using the ggplot2 package (accessed on 25 April 2024). Permutational multivariate analysis of variance (PERMANOVA) was used to test significant differences in assemblage composition between sampling methods and among phenological periods (pre-flowering, flowering and post- flowering) of *T. cordata*.

Indicator taxa associated with phenological periods (before, during, and after T. cordata flowering) were identified using Multilevel Pattern Analysis (MPA) with the multipatt function from the indicspecies package in R, with 999 permutations (accessed on 5 June 2023). Results of the MPA are presented in the Supplementary Material (Table S3). For all inferential tests, we report the corresponding test statistics and effect size measures alongside *p*-values (Mann–Whitney–Wilcoxon W, Kruskal–Wallis H, PERMANOVA F and R^2^). For NMDS ordinations, two-dimensional stress values are provided to assess ordination quality.

## 3. Results

Across all sites and both years (2023–2024), net sampling yielded 5045 insects representing 207 taxa, whereas sticky traps captured 44,851 insects representing 86 taxa. In net samples, 2900 individuals (57.5% of the net total) comprising 172 taxa were collected in 2023, and 2145 individuals (42.5%) comprising 147 taxa in 2024. In sticky traps, 24,901 individuals (55.5% of the sticky-trap total) comprising 72 taxa were collected in 2023, and 19,950 individuals (44.5%) comprising 60 taxa in 2024 (Table 2).

The Kruskal–Wallis test showed that species richness was significantly higher in net samples than in sticky traps (*H* = 12.8, *p* = 0.0003), while total abundance was significantly higher in sticky traps than in net samples (*H* = 6.2, *p* = 0.013). Within each method, neither species richness nor total abundance differed significantly between years (2023 vs. 2024; *p* > 0.05), and Shannon diversity did not differ between 2023 and 2024 for either net samples (*H* = 3.56 vs. 3.66) or sticky traps (*H* = 2.06 vs. 1.92). When both years and all sites were combined, the Mann–Whitney–Wilcoxon test indicated that Shannon diversity was significantly higher in net samples than in sticky traps (*H* = 3.81 vs. 2.10; *W =* 71, *p* = 0.0005) (Table 2). Overall, net sampling revealed higher species richness and diversity, whereas sticky traps yielded higher total relative abundance.

**Figure 2:**
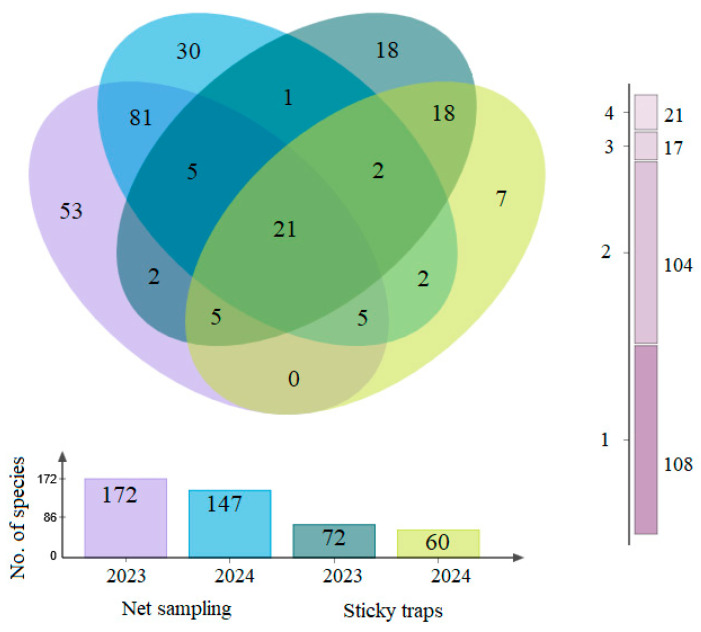
Venn diagram showing the numbers of unique and shared insect taxa between net sampling and sticky traps in *Tilia cordata* GCUs in 2023 and 2024. The vertical scale indicates the total number of unique species (1), and shared species (2, 3 and 4). For each method and year, data from different sites are combined.

Across methods, 21 taxa were shared between the net sampling and sticky trap datasets during 2023–2024. In net samples (207 taxa in total), 53 occurred only in 2023, 30 only in 2024, and 81 were found in both years (Figure 2).

In the sticky trap samples (86 taxa in total), 18 occurred only in 2023, 7 only in 2024, and 18 were found in both years (Figure 2).

**Figure 3:**
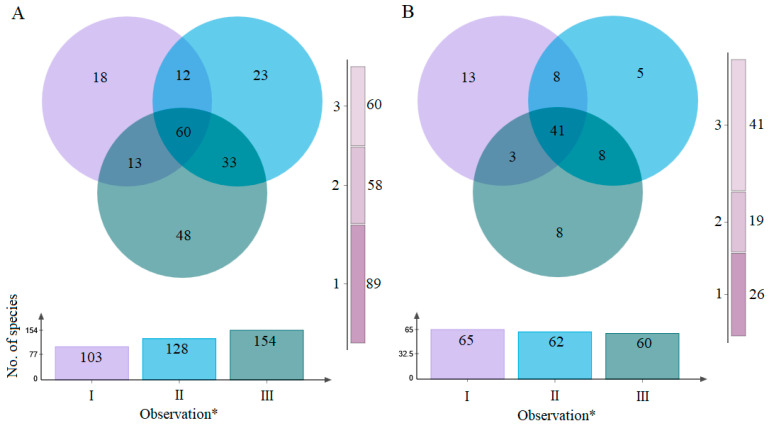
Venn diagram showing numbers of unique and shared insect taxa among phenological periods (Observation *) of *Tilia cordata* pre-flowering (I); flowering (II); and post-flowering (III). Net sampling (**A**); sticky traps (**B**). The vertical scale indicates the total number of unique species (1), and shared species (2 and 3). For both methods, data from different sites and both years are combined.

When data from both years and all sites were combined,18 taxa in net samples were unique to the *T. cordata* pre-flowering period (I), 23 to flowering (II), and 48 to post-flowering (II), while 60 occurred in all periods (Figure 3A).

In the sticky trap samples, 13 taxa were unique to pre-flowering (I), 5 to flowering (II), and 8 to post-flowering (II), while 41 taxa were shared across all periods (Figure 3B).

**Figure 4:**
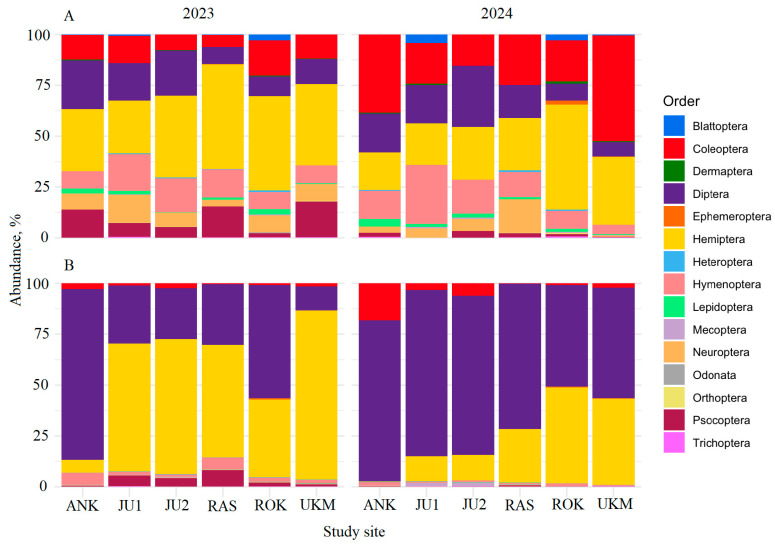
Relative abundance of insect orders collected by net sampling (**A**) and sticky traps (**B**) at different sites within *Tilia cordata* GCUs in 2023 and 2024.

Across 2023–2024, 15 insect orders were recorded in net samples. The most common were Hemiptera (35.3%), followed by Coleoptera (20.8%), Diptera (15.1%), and Hymenoptera (12.1%). Sticky trap samples contained 11 insect orders, dominated by Diptera (50.2%) and Hemiptera (41.9%) when all study sites and both years were combined (Figure 4A,B). In net samples, Hemiptera were more abundant in 2023 than in 2024 (39.7% vs. 29.2%; *F* = 4.5, *p* < 0.05 across sites), whereas Coleoptera was more abundant in 2024 than in 2023 (33.6% vs. 11.3%; *F* = 5.2, *p* < 0.05) Other insect orders in the net samples showed no significant between-year differences (*F* = 2.0, *p* > 0.05) (Figure 4A). In sticky trap samples, Diptera were more abundant in 2024 than in 2023 (67.6% vs. 36.2%; *F* = 16.7, *p* < 0.05), while Hemiptera were more abundant in 2023 than in 2024 (54.9% vs. 25.5%; *F* = 20.4, *p* < 0.05). For the remaining, less abundant orders in sticky traps, no significant between-year differences were detected (*F* = 2.5; *p* > 0.05) (Figure 4B).

Across 2023–2024, net samples were dominated by a small number of taxa: *Empoasca vitis* (17.7% of all individuals), *Meligethes aeneus* (6.4%), *Caecilius flavidus* (6.1%), *Lauxaniidae* sp. 1 (5.0%), and *Ichneumonidae* sp. 4 (4.2%) (Table 3). The identity and relative importance of dominant taxa shifted between phenological periods and between years, whereas the remaining taxa among the 10 most common species occurred at lower abundances and varied idiosyncratically across periods and years (Table 3).

Sticky trap samples showed a similar pattern of dominance by a few taxa (Table 3). Across 2023–2024, *E. vitis* (33.9% of all individuals), *Mycetophilidae* sp. 1 (27.6%), and *Diptera* sp. 1 (10.1%) were most abundant. Their relative abundances varied between phenological periods and between years, but taken together, they consistently accounted for a large fraction of the sticky-trap catch, while the remaining taxa among the 10 most common species occurred at comparatively low and variable abundances (Table 3).

**Figure 5:**
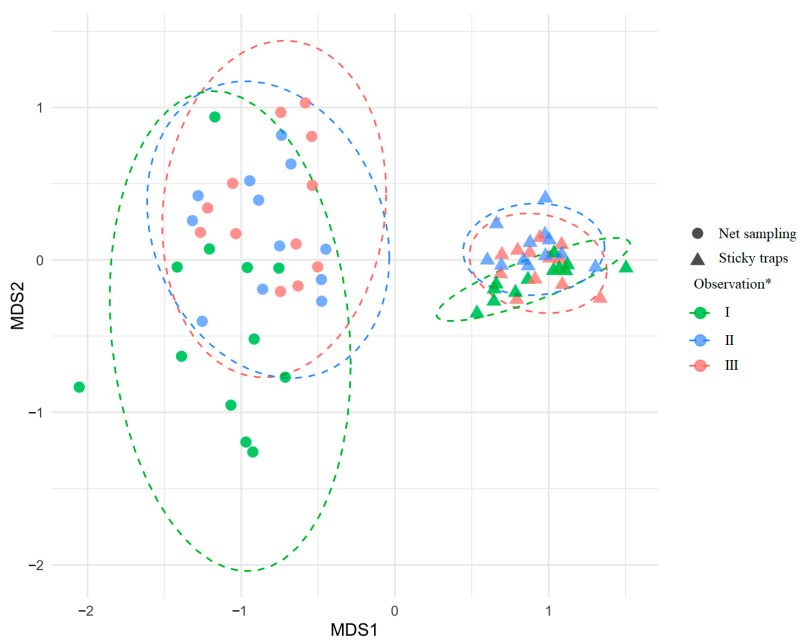
NMDS ordination diagram (Stress = 0.13) based on nonmetric multidimensional scaling of insect assemblages collected by net sampling and sticky traps across phenological periods (Observation *): pre-flowering (I), flowering (II), and post-flowering (III) of *Tilia cordata.* Data from different *T. cordata* GCUs and both years are combined.

Non-metric multidimensional scaling (NMDS, Stress = 0.13) revealed a clear separation between insect assemblages captured by net sampling and sticky traps (Figure 5). Within each method, assemblages from the three phenological periods of *T. cordata* partly overlapped. Net samples showed greater dispersion in ordination space, indicating higher among-sample variability, whereas sticky trap samples formed tighter clusters, indicating more homogeneous assemblages (Figure 5). PERMANOVA confirmed these patterns, detecting significant differences between methods (*F* = 38.9, *R*^2^ = 0.32, *p* = 0.001) and significant temporal variation across periods I–III (*p* = 0.002).

Multilevel Pattern Analysis (MPA) pooled across years identified 41 taxa significantly associated with net sampling and 28 with sticky traps. In the net samples, 17, 41, and 51 taxa were significantly associated with pre-flowering (I), flowering (II), and post-flowering (III), respectively, whereas in the sticky trap samples, 29, 32, and 29 taxa were significantly associated with these periods. Indicator taxa largely reflected the most abundant and consistently recorded components of the assemblages (e.g., *E. vitis*, *Lauxaniidae* sp. 1, *E. tiliae*, *Mycetophilidae* sp. 1, *Diptera* sp. 1). Full lists of indicator taxa, indicator values, and *p*-values are provided in Supplementary Table S3.

**Figure 6:**
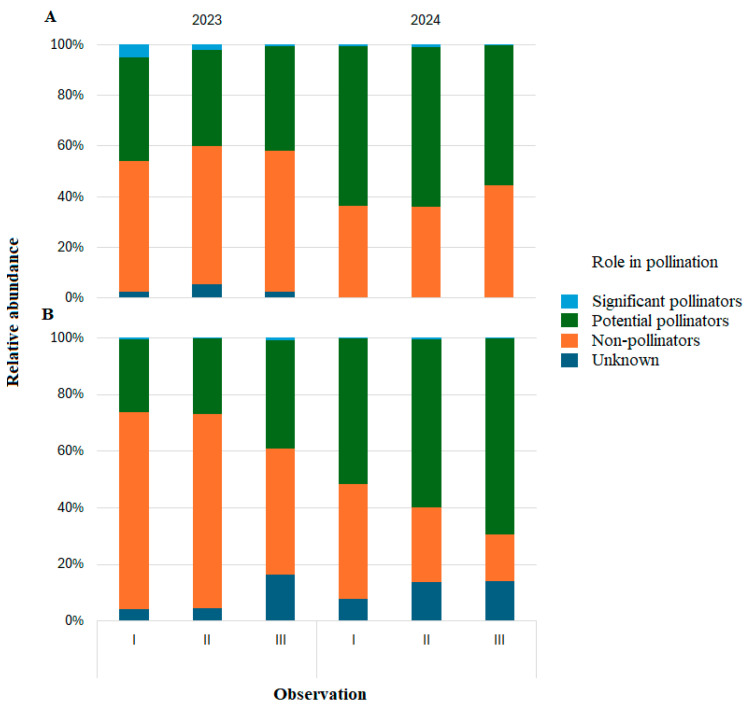
Assignment of insects to pollination-related categories in net samples (**A**) and sticky trap samples (**B**), across observation periods, pre-flowering (I), flowering (II), and post-flowering (III), of *Tilia cordata.* Data from different *Tilia. cordata* GCUs are combined.

When insects were analyzed according to their role in pollination, net samples contained only low proportions of taxa classified as significant pollinators, accounting for <5% of individuals. Their relative abundances fluctuated among three phenological periods and between years, with no consistent association with the *T. cordata* flowering period. Potential pollinators consistently comprised about 40–60% of the net collected insects, with similar proportions across observation periods (I–III) in both years, likewise showing no clear relationship with the flowering phase of *T. cordata* (Figure 6A).

In sticky trap samples, insects classified as potential pollinators comprised between 25 and 70% of individuals, with stronger variation in abundance across all periods and in both years. Significant pollinators comprised only about 1% of all collected insects. The remaining 30–74% were classified as non-pollinators or as having an unknown role in pollination. Their proportions also varied without consistent trends across periods or years (Figure 6B).

**Figure 7:**
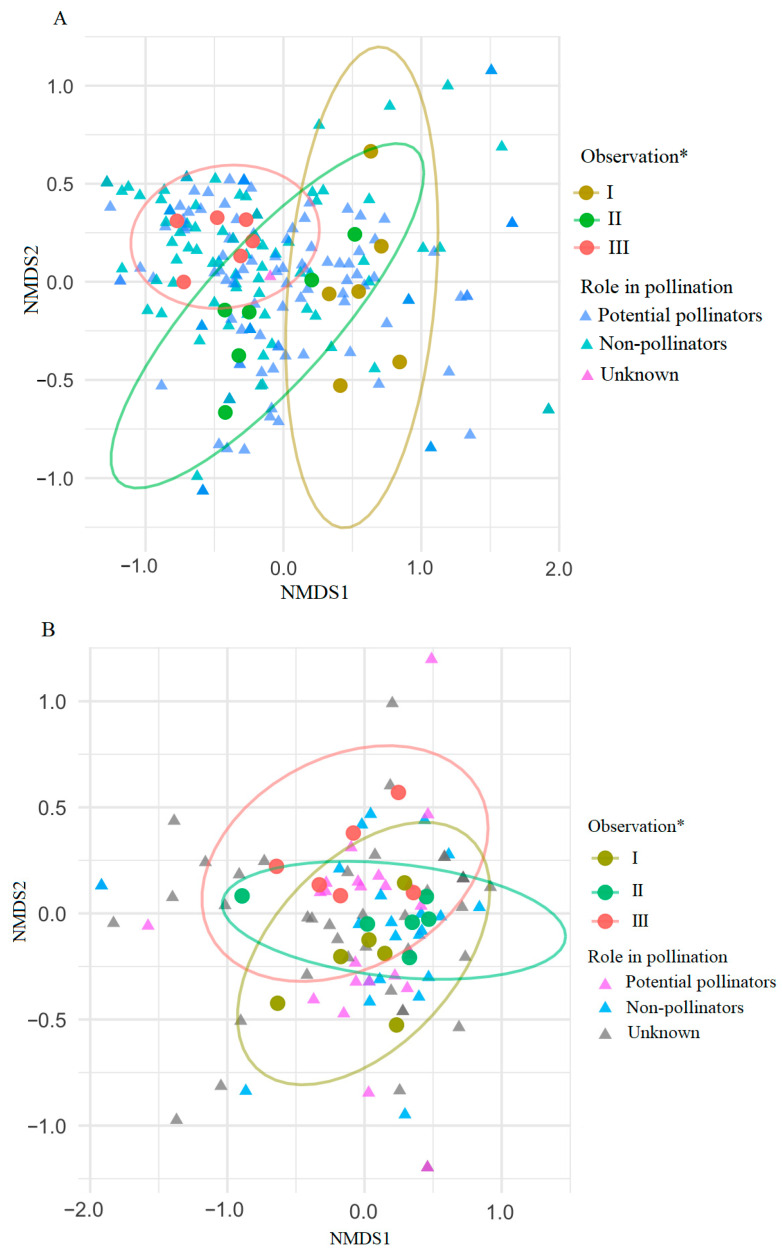
NMDS ordination diagrams based on nonmetric multidimensional scaling of insect assemblages categorized by pollination role. Net samples (**A**), (Stress = 0.15) and sticky traps (**B**), (Stress = 0.12), across periods of observation *: pre-flowering (I), flowering (II), and post-flowering (III) of *Tilia cordata.* Potential pollinators include significant pollinators. Data from different *T. cordata* GCUs and both years are combined.

In the net samples, NMDS (Stress = 0.15) based on pollination role showed substantial overlap among the three observation periods, indicating broadly similar insect assemblage composition throughout pre-flowering, flowering and post-flowering of *T. cordata*. Taxa classified as potential pollinators, non-pollinators, and taxa with unknown pollination roles were intermixed across ordination space, with potential pollinators forming a somewhat tighter cluster near the center and non-pollinators plus unknowns being more widely dispersed (Figure 7A). This suggests that variation in insect assemblage structure at the study sites is modest and not strongly structured by pollination role.

Similarly, in sticky trap samples, the NMDS ordination (Stress = 0.12) showed considerable overlap among observation periods (I–II), indicating only minor temporal changes in insect assemblage structure. Potential pollinators, non-pollinators, and insects with unknown pollination roles were interspersed across ordination space, again indicating weak functional segregation (Figure 7B). Together, these patterns indicate that both temporal variation and pollination role play only a minor role in structuring the observed insect assemblages.

## 4. Discussion

### 4.1. Methodological Effect on Insect Diversity and Assemblages

Over two years, we monitored insect assemblages associated with *T. cordata* using net sampling and yellow sticky traps, two widely applied but methodologically contrasting approaches [44]. Consistent with known color biases [45], yellow traps mainly captured flying insects attracted to yellow, particularly Diptera, but yielded few taxa classified as significant or potential pollinators and many non-pollinators or taxa with unknown pollination roles (Figure 6B). This matches earlier findings that color-based traps do not selectively sample pollinators and are unsuitable for inferring pollination effectiveness or interaction importance [46,47,48,49,50]. Net sampling, by contrast, produced a higher proportion of taxa classified as potential or significant pollinators (Figure 6A). As emphasized by Thompson et al. [44], active sampling from the flowering vegetation layer provides more direct information on plant–insect associations. In our study, netting at 1–2 m height targeted the lower canopy and flowering strata of *T. cordata* and thus better represented active flower visitors. Despite this, significant pollinators (e.g., wild bees) remained scarce, likely due to the protected forest interior context, distant from urban plantings and mass-flowering crops and lacking external floral subsidies [27,51,52]. These habitat conditions probably limited both diversity and local activity of significant pollinators in *T. cordata* GCUs. Net samples exhibited higher insect diversity and richness, whereas sticky traps yielded much higher total abundance (Table 2). This aligns with evidence that active methods capture a broader taxonomic spectrum, including behaviorally specialized and visually oriented flower visitors [53,54], while sticky traps accumulate large numbers of small, abundant taxa over longer exposures, inflating abundance but lowering diversity [53]. NMDS ordination showed clear compositional separation between methods, with more homogeneous sticky trap assemblages and more heterogeneous net assemblages (Figure 6), consistent with strong method effects, whereby passive traps yielded more uniform samples dominated by highly catchable taxa [46]. Overall, net sampling better characterized active insect assemblages in the flowering strata of *T. cordata*, whereas sticky traps mainly captured a numerically dominant but functionally mixed subset of the flying fauna. Part of these methodological contrasts may also reflect vertical stratification and habitat context (lower-canopy edges vs. mid-canopy forest interiors), so method differences should be interpreted cautiously.

### 4.2. Temporal and Spatial Effects on Insect Assemblage Composition

Insect communities from pre-flowering, flowering, and post-flowering periods partly overlapped, although PERMANOVA detected significant temporal variation (Figure 5), similar to other studies showing temporal shifts with substantial overlap [55,56]. We found no distinct peak in insect abundance or species richness during *T. cordata* flowering. Diversity and dominance patterns were broadly similar across phenological phases, and potential pollinators, non-pollinators and taxa with unknown roles were intermixed in all periods (Figure 7A,B). These results support the view that *Tilia* spp. act as generalized, mass-flowering resources attracting a broad taxonomic and functional spectrum of visitors [8,57]. Dense aphid populations producing honeydew on *Tilia* spp. further attract diverse insects (Diptera, Hymenoptera, Coleoptera) independently of flowering, mixing pollinators and non-pollinators in space and time, and weakening structuring by pollination role [58,59,60]. Across 2023–2024, we recorded 15 insect orders in net samples, dominated by Hemiptera, Coleoptera, Diptera and Hymenoptera, while sticky traps were dominated by Diptera and Hemiptera (Figure 4A,B). These groups are among the most species-rich and abundant in terrestrial ecosystems and commonly dominate forest and flower-associated assemblages [27,61,62,63,64,65].

Year-to-year variation in dominant orders and differences among *T. cordata* GCUs (Figure 4A,B) likely reflect interannual climatic variation and site-specific differences in host phenology, flowering intensity and honeydew production. Even modest changes in temperature and precipitation can shift order-level abundances via effects on survival, voltinism, diapause and resource availability [61,62,66]. Spatial variation in the timing and intensity of host–plant phenology also structures canopy-dwelling and flower-visiting assemblages by altering which taxa are active at given sampling dates [67,68,69]. Variation in flowering intensity of *Tilia* spp. can modify visitation rates and visitor guild composition [70,71]. The lack of a pronounced diversity or abundance peak during flowering likely reflects: (i) closed-canopy interiors with sparse herbaceous flowering layers can support lower densities of specialized floral visitors [52]; (ii) prolonged honeydew availability from aphids on *Tilia* spp., sustaining insects before, during and after flowering [58,59,60]; and (iii) our focus on a single canopy layer with monthly sampling, which may have missed brief visitation peaks tied to short flowering maxima or favorable weather conditions.

### 4.3. Species Richness and Possible Host Association

Across 2023–2024, we recorded 247 insect taxa in *T. cordata* GCUs (Table 2), comparable to or slightly higher than values from similar temperate forests using analogous trapping schemes (ca. 150–300 taxa per stand over 1–3 years [72,73,74]). Overlap between methods and between years was low (Figure 2), indicating that each method sampled largely distinct subsets, whereas within-method overlap among phenological periods was higher (Figure 3A,B). This underscores strong methodological effects and supports using multiple approaches to characterize forest insect assemblages [49]. Community structure in both approaches was shaped by a few abundant taxa and many rare species (Table 3), a common pattern in forest canopies [75,76,77]. Only a limited number of taxa reached high relative abundances, with dominance varying between years and phenological phases and showing no clear dependence on flowering. *Empoasca vitis* was particularly abundant in both method samples across years and phenological phases (Table 3), consistent with its ecology as a highly mobile, polyphagous leafhopper associated with numerous woody and herbaceous hosts [78,79] and paralleling the prevalence of vagile, polyphagous herbivores in other forest canopies [80]. *Eucallipterus tiliae*, a numerically dominant aphid specialized on *Tilia* spp. [81,82,83], was also frequent (Table 3), reflecting a strong herbivore-host association. Several other abundant taxa appeared more linked to forest structure and microhabitat than specifically to *T. cordata*. *Meligethes aeneus*, a Brassicaceae specialist and major oilseed rape pest with only occasional records on trees [84], *Caecilius flavidus*, associated with trunks and branches of broad-leaved trees, including *Tilia* spp. [85], and saprophagous lauxaniid flies of shaded woodlands [86] exemplify this pattern. We also examined insect taxa associated with pre-flowering, flowering and post-flowering. MPA showed a small, largely overlapping set of taxa significantly associated with flowering and post-flowering in net samples, while dominant sticky-trap taxa were similar across all phenological stages (Table S3). No taxa were exclusively associated with *T. cordata* flowers. This weak flowering-phase specificity suggests that dominant taxa were driven more by broader host-plant associations, local microclimate [87], landscape context and forest management regimes [88] than by *T. cordata* floral resources alone.

### 4.4. Possible Pollination Roles of Recorded Insect Taxa

Assigning insect taxa to functional pollination roles is difficult, especially for non-bee groups often identified only to order or family [18,89]. We recorded a broad spectrum of flower-associated insects, including Diptera, Coleoptera and Hymenoptera. Several taxa known as effective pollinators were present in both method samples, but at low abundances. Within Diptera, we identified Syrphidae such as *Episyrphus balteatus*, *Melanostoma scalare*, *Scaeva pyrastri* and *Brachypalpoides lentus* (Tables S1 and S2), recognized as important pollinators in temperate ecosystems [90,91,92]. Among Coleoptera, taxa from Cantharidae and Melyridae were recorded (Tables S1 and S2). These beetle families include pollen-feeding species acting as incidental pollinators in forests and agroecosystems [18,21,93]. Within Hymenoptera, *Apis mellifera* and *Megachilidae* spp. (Tables S1 and S2) represent significant pollinators in woody and herbaceous flora [25,94]. Many other taxa have primary ecological roles other than pollination (parasitoids, predators, saprophages, herbivores, fungivores) but may still act as incidental pollinators via opportunistic flower visitation [95]. Several taxa in our material can therefore be conservatively treated as flower visitors with potential but unquantified contributions in *T. cordata* stands. Ants illustrate this dual role. *Lasius niger* and *Myrmica rubra* are generalists visiting flowers and honeydew [96,97,98]. Although often considered suboptimal pollinators due to smooth integument and antimicrobial secretions, they can transport pollen and are regarded as facultative pollinators in some systems [98]; their presence in *T. cordata* GCUs (Table 3, Tables S1 and S2) fits this classification. Green lacewings (Chrysopidae) show a similar pattern: predatory larvae but adults feeding on pollen, nectar and honeydew [99]. Adult *C. perla* and *C. adspersa* are recorded on inflorescences in field margins, where feeding on pollen and nectar has been documented [100]. These lacewings can incidentally transfer pollen. Parasitic wasps (Ichneumonidae, Chalcidoidea) often use floral nectar as adults, enhancing longevity and reproduction [101,102]. *Pteromalus puparum* and *Pimpla* sp. 1, present in our samples (Tables S1 and S2), fit this pattern, though species-specific pollination data are lacking. Tachinid flies (e.g., *Peleteria* spp.) and horseflies (*Tabanus bovinus*) also feed on nectar when not blood-feeding, and several tabanids are regular flower visitors and incidental pollinators [64,103]. Overall, *T. cordata* stands host a taxonomically and functionally diverse but generally low-abundance assemblage of flower visitors extending beyond bees to beetles, hoverflies, ants, lacewings and parasitoid wasps. For most taxa, pollination contributions are probably incidental and unquantified. Our data do not support viewing *T. cordata* in forest interiors as a keystone melliferous resource. Rather, *T. cordata* seems to sustain a broad flower-associated insect fauna, while effective pollination likely depends on a small subset of specialized or efficient pollinators at low densities [18,104]. As we did not directly measure pollination effectiveness (pollen loads, visitation rates, fruit/seed set), our inferences rely on presence, assemblage structure and literature-based functional assignments rather than experimentally demonstrated pollination.

## 5. Concluding Remarks

Overall, *T. cordata* stands within protected areas supported relatively high insect diversity, encompassing recognized pollinators as well as a wide spectrum of incidental flower visitors. However, only a small fraction of the recorded taxa could be regarded as important pollinators, and their abundances were generally low.

In the studied forest GCUs, *T. cordata* does not act as a keystone melliferous species for pollinators, but rather as one of several resources sustaining a taxonomically diverse, functionally mixed assemblage of flower-associated insects. Temporal variation in assemblage composition and pollination role was modest, and assemblages showed substantial overlap among pre-flowering, flowering, and post-flowering periods.

Empirical data on potential pollinators in forest ecosystems, including *T. cordata* stands, remain limited. Future research should adopt a trait-based, integrative framework that explicitly incorporates non-bee taxa, links floral visitation to functional traits and pollen transfer, and evaluates how forest management, landscape context, and protection status shape the diversity and effectiveness of these assemblages. Such an approach will be essential for developing realistic, ecosystem-scale assessments of pollination in forest habitats and for recognizing the often modest, but potentially complementary contributions of non-bee insects to pollination processes in temperate forests. Overall, our data documents the presence of a diverse assemblage of potential pollinators associated with *T. cordata*, but they do not allow us to quantify realized pollination services or to demonstrate causal links between particular taxa and effective pollination of *T. cordata* in these forest stands.

## Figures and Tables

**Table 1:** Characteristics of *Tilia cordata* GCUs and study sites. Information is based on the forest inventory data obtained from the State Forest Cadastre as of 1 March 2023.

Site	Geographical Position	GCU Size (ha)	Age (y)	Mean Height (m)	Mean Diameter (cm)	Forest Site Type *	Forest Vegetation Type **	Tree Species Composition (%) ***
ANK	55°32′12.0228″ N, 24°53′ 30.1014″ E	1.84	79	27.4	28.8	Lcs	mox	60T 30P 10B
JU1	55°10′5.7714″ N, 23°19′45.7134″ E	3.8	138	28.3	40.0	Lfp	aeg	60T 20S10Q 10A
JU2	55°9′28.6272″ N, 23°19′32.16″ E	5.32	93	25.6	33.6	Lfp	aeg	70T 20F 10S
RAS	55°20′25.029″ N, 23°38′48.1662″ E	7.23	98	26.6	41.7	Lds	aeg	40T 20Q 20B 20S
ROK	55°47′56.331″ N, 25°48′24.8178″ E	2.87	84	26.3	30.8	Lds	aeg	70T 10B 20S
UKM	54°58′5.9118″ N, 25°11′8.7102″ E	20.23	94	29.3	34.8	Lds	aeg	50T 40P 10Q

* L: temporarily waterlogged mineral soils; c: moderate fertility; d: high fertility; f: very high fertility.; s: heavy soil texture; p: two-layered soil structure with a light fraction on a heavy fraction or vice versa [31]. ** aeg: *aegopodiosa*; mox: *myrtillio-oxalidosa* [32]. *** T: *Tilia cordata*; Q: *Quercus robur*; B: *Betula pendula*; S: *Picea abies*; P: *Populus tremula*; A: *Acer platanoides*; F: *Fraxinus excelsior*. In each stand, tree species composition is based on the volume.

**Table 2:** Relative abundance (%), species richness (%) and Shannon (*H*) diversity of insects collected by net sampling and sticky traps at *Tilia cordata* GCUs in 2023 and 2024.

Site	2023			2024			Both Year		
	Relative Abundance, % (No. of Individuals)	Species Richness, % (No. of Species)	Shannon *H*	Relative Abundance, % (No. of Individuals)	Species Richness, % (No. of Species)	Shannon *H*	Relative Abundance, % (No. of Individuals)	Species Richness, % (No. of Species)	Shannon *H*
Net Sampling									
ANK	18.1 (526)	39.0 (67)	3.20	22.0 (471)	43.5 (64)	3.26	19.8 (997)	48.3 (100)	3.52
JU1	14.7 (427)	48.3 (83)	3.57	8.9 (190)	32.7 (48)	3.24	12.2 (617)	50.7 (105)	3.75
JU2	13.4 (388)	37.8 (65)	3.17	9.4 (202)	29.9 (44)	3.15	11.7 (590)	40.1 (83)	3.37
RAS	21.4 (621)	36.6 (63)	2.53	18.6 (400)	37.4 (55)	3.17	20.2 (1021)	44.9 (93)	3.09
ROK	17.5 (507)	50.6 (87)	3.59	12.7 (273)	39.5 (58)	3.26	15.4 (779)	51.7 (107)	3.66
UKM	14.9 (432)	40.1 (69)	3.06	28.4 (609)	41.5 (61)	2.77	20.6 (1041)	47.8 (99)	3.30
All sites	100.0 (2900)	100.0 (172)	3.56	100.0 (2145)	100.0 (147)	3.66	100.0 (5045)	100.0 (207)	3.81
Year proportion, %	57.5	83.1	-	42.5	71.0	-	100	100	-
Sticky traps									
ANK	12.6 (3129)	54.2 (39)	1.52	14.6 (2915)	51.7 (31)	1.54	13.5 (6044)	55.8 (48)	1.65
JU1	18.3 (4548)	59.7 (43)	1.98	14.4 (2865)	61.7 (37)	2.03	16.5 (7413)	62.8 (54)	2.29
JU2	20.4 (5087)	59.7 (43)	1.83	14.4 (2876)	55.0 (33)	1.83	17.8 (7963)	58.1 (50)	2.16
RAS	17.2 (4286)	56.9 (41)	1.93	18.0 (3596)	55.0 (33)	1.45	17.6 (7882)	54.7 (47)	1.84
ROK	14.8 (3695)	51.4 (37)	1.75	16.6 (3315)	55.0 (33)	1.80	15.6 (7010)	54.7 (47)	1.82
UKM	16.7 (4156)	62.5 (45)	1.32	22.0 (4383)	55.0 (33)	1.58	19.0 (8539)	64.0 (55)	1.67
All sites	100.0 (24,901)	100.0 (72)	2.06	100.0 (19,950)	100.0 (60)	1.92	100.0 (44,851)	100.0 (86)	2.10
Year proportion, %	55.5	83.7	-	44.5	69.8	-	100.0	100.0	-
All total							49,896 (100.0)	247 (100.0)	

**Table 3:** Relative abundance (%) of the most common 10 insect taxa collected by net sampling and sticky traps across phenological periods in 2023-2024. Data from different *Tilia cordata* GCUs are combined.

Order	Family	Insect Taxa	2023				2024				Both Year
			Observation *								
			I	II	III	Total	I	II	III	Total	
			Net Sampling								
Hemiptera	Cicadellidae	*Empoasca vitis*	4.9	25.1	24.4	22.5	17.1	11.0	8.4	11.2	17.7
Coleoptera	Nitidulidae	*Meligethes aeneus*	1.0	1.3	0.1	0.5	0.3	28.8	0.1	14.3	6.4
Psocoptera	Caeciliusidae	*Caecilius flavidus*	27.0	4.6	9.6	9.9	3.2	0.1	0.7	0.8	6.1
Diptera	Lauxaniidae	*Lauxaniidae* sp. 1	6.2	7.2	4.1	5.2	13.3	3.5	2.1	4.8	5.0
Hymenoptera	Ichneumonidae	*Ichneumonidae* sp. 4	5.2	3.9	5.1	4.8	3.2	2.5	5.3	3.5	4.2
Hemiptera	Aphididae	*Eucallipterus tiliae*	0.7	8.7	1.5	3.6	0.3	4.4	5.0	3.9	3.7
Hymenoptera	Campanotidae	*Lasius niger*	3.6	4.8	1.2	2.6	12.8	2.1	3.4	4.4	3.3
Neuroptera	Chrysopidae	*Chrysopa perla*	1.0	1.3	5.8	3.9	-	-	5.3	1.8	3.0
Coleoptera	Helodidae	*Cyphon padi*	0.7	0.3	0.1	0.2	6.1	10.5	0.6	6.4	2.9
Neuroptera	Chrysopidae	*Chrysopa adspersa*	-	0.1	4.5	2.7	-	0.9	7.0	2.8	2.8
Total of 10 species			50.2	57.3	56.4	56.0	56.3	63.7	38.0	53.9	55.1
			Sticky traps								
Hemiptera	Cicadellidae	*Empoasca vitis*	48.8	49.3	35.4	43.7	33.2	18.8	6.9	21.7	33.9
Diptera	Mycetophilidae	*Mycetophilidae* sp. 1	15.6	17.0	17.1	16.7	39.3	45.7	40.3	41.2	27.6
Diptera	Unknown	*Diptera* sp. 1	4.3	4.5	16.5	9.2	7.7	13.8	14.2	11.2	10.1
Hemiptera	Aphididae	*Eucallipterus tiliae*	4.9	15.8	2.3	8.2	0.3	1.7	3.4	1.6	5.3
Diptera	Muscidae	*Musca domestica*	1.7	2.4	4.3	3.0	4.5	8.0	11.3	7.4	5.0
Diptera	Mycetophilidae	*Mycetophilidae* sp. 2	0.3	0.6	10.2	4.3	-	-	13.5	4.0	4.2
Psocoptera	Caecillidae	*Caecilius flavidus*	12.2	0.7	1.4	3.4	-	0.1	0.5	0.2	2.0
Coleoptera	Helodidae	*Cyphon variabilis*	-	-	0.3	0.1	5.5	3.6	0.1	3.4	1.6
Diptera	Tachinidae	*Peleteria* sp. 1	0.8	0.4	0.6	0.6	3.9	1.5	1.4	2.5	1.4
Hemiptera	Cicadellidae	*Anaceratagallia ribauti*	<0.1	0.1	2.9	1.2	<0.1	0.7	5.0	1.7	1.4
Total of 10 species			88.6	90.8	90.6	90.4	94.4	93.7	94.0	96.4	94.9

Observation *: phenological periods of *T. cordata* pre-flowering (I); flowering (II); and post-flowering (III).

## Data Availability

The original contributions presented in this study are included in the article/Supplementary Material. Further inquiries can be directed to the corresponding author.

## Supplementary Materials

The following supporting information can be downloaded at: https://www.mdpi.com/article/10.3390/insects17040360/s1, Table S1: Relative abundance (%) functional role of insect taxa detected by net sampling in different observation periods: *Tilia cordata* pre-flowering (I), flowering (II) and post-flowering in 2023–2024; Assignation by insect functional role, refs. [21,83,95,96,97,101,102,103,105,106,107,108,109,110,111,112,113,114,115,116,117,118,119,120,121,122,123,124,125,126,127,128,129,130,131,132,133,134,135,136,137,138,139,140,141,142,143,144,145,146,147,148,149,150,151,152,153,154,155,156,157,158,159,160,161,162,163,164,165,166,167,168,169,170,171,172,173,174,175,176,177,178,179,180,181,182,183,184,185,186,187,188,189,190,191,192,193,194,195,196,197,198,199,200,201,202,203,204,205,206,207,208,209,210,211,212,213,214,215,216,217,218,219,220,221,222,223,224,225]; Table S2: Relative abundance (%) and functional role of insect taxa detected by the sticky traps in different observation periods: *Tilia cordata* pre-flowering (I), flowering (II) and post-flowering in 2023–2024; Assignation by insect functional role, refs. [21,83,95,101,105,107,108,109,110,111,112,114,115,116,117,120,122,124,125,127,131,133,137,138,145,146,151,152,153,154,155,161,168,176,182,188,193,196,197,210,221,225,226,227,228,229,230,231,232,233,234,235,236,237,238]; Table S3: Association among insect taxa and sampling method (net sampling and sticky traps) and periods of *Tilia cordata* pre-flowering, flowering and post-flowering according to Multilevel Pattern Analysis (MPA of indicator species), significance level (alpha): 0.05.
